# Multipotent adult germ-line stem cells, like other pluripotent stem cells, can be killed by cytotoxic T lymphocytes despite low expression of major histocompatibility complex class I molecules

**DOI:** 10.1186/1745-6150-4-31

**Published:** 2009-08-28

**Authors:** Ralf Dressel, Kaomei Guan, Jessica Nolte, Leslie Elsner, Sebastian Monecke, Karim Nayernia, Gerd Hasenfuss, Wolfgang Engel

**Affiliations:** 1 Department of Cellular and Molecular Immunology, University of Göttingen, Heinrich-Düker-Weg 12, 37073 Göttingen, Germany; 2 Department of Cardiology and Pneumology, University of Göttingen, Robert-Koch-Str. 40, 37075 Göttingen, Germany; 3 Institute of Human Genetics, University of Göttingen, Heinrich-Düker-Weg 12, 37073 Göttingen, Germany; 4 North East Stem Cell Institute and Institute of Human Genetics, University of Newcastle upon Tyne, International Centre for Life, Central Parkway, Newcastle upon Tyne NE1 3BZ, UK

## Abstract

**Background:**

Multipotent adult germ-line stem cells (maGSCs) represent a new pluripotent cell type that can be derived without genetic manipulation from spermatogonial stem cells (SSCs) present in adult testis. Similarly to induced pluripotent stem cells (iPSCs), they could provide a source of cellular grafts for new transplantation therapies of a broad variety of diseases. To test whether these stem cells can be rejected by the recipients, we have analyzed whether maGSCs and iPSCs can become targets for cytotoxic T lymphocytes (CTL) or whether they are protected, as previously proposed for embryonic stem cells (ESCs).

**Results:**

We have observed that maGSCs can be maintained in prolonged culture with or without leukemia inhibitory factor and/or feeder cells and still retain the capacity to form teratomas in immunodeficient recipients. They were, however, rejected in immunocompetent allogeneic recipients, and the immune response controlled teratoma growth. We analyzed the susceptibility of three maGSC lines to CTL in comparison to ESCs, iPSCs, and F9 teratocarcinoma cells. Major histocompatibility complex (MHC) class I molecules were not detectable by flow cytometry on these stem cell lines, apart from low levels on one maGSC line (maGSC Stra8 SSC5). However, using a quantitative real time PCR analysis *H2K *and *B2m *transcripts were detected in all pluripotent stem cell lines. All pluripotent stem cell lines were killed in a peptide-dependent manner by activated CTLs derived from T cell receptor transgenic OT-I mice after pulsing of the targets with the SIINFEKL peptide.

**Conclusion:**

Pluripotent stem cells, including maGSCs, ESCs, and iPSCs can become targets for CTLs, even if the expression level of MHC class I molecules is below the detection limit of flow cytometry. Thus they are not protected against CTL-mediated cytotoxicity. Therefore, pluripotent cells might be rejected after transplantation by this mechanism if specific antigens are presented and if specific activated CTLs are present. Our results show that the adaptive immune system has in principle the capacity to kill pluripotent and teratoma forming stem cells. This finding might help to develop new strategies to increase the safety of future transplantations of *in vitro *differentiated cells by exploiting a selective immune response against contaminating undifferentiated cells.

**Reviewers:**

This article was reviewed by Bhagirath Singh, Etienne Joly and Lutz Walter.

## Background

Pluripotent stem cells could provide the basis for restoring tissue functions by transplantation of cellular grafts in a broad variety of diseases. Embryonic stem cells (ESCs) are the best characterized pluripotent stem cells. They clearly have the capacity to self-renew and to give rise to any cell type of the body [1]. However, the use of human ESCs is restricted due to severe ethical concerns. Therefore, new pluripotent cell types that are derived from adult organisms hold great promises for regenerative medicine. Recently, it has been shown that pluripotent cells can be obtained from spermatogonial stem cells (SSCs) of neonatal [2] and even adult mice [3-5]. Although SSCs are known to be unipotent [6] they can undergo reprogramming during in vitro cultivation and give rise to multipotent adult germ-line stem cells (maGSCs) that can differentiate into various cell types *in vitro*, and form teratomas *in vivo *[3,7]. The maGSCs have significant similarities to ESCs [8,9] and their differentiation capacity into functional cardiomyocytes [10] and neuronal cells [11,12] has been shown. Therefore, maGSCs are expected to have a high potential for regenerative medicine [13]. The generation of pluripotent cells from adult human testis has been recently reported [14-16]. Since maGSCs can be obtained without the need of genetic manipulation, they might have advantages even compared to induced pluripotent stem cells (iPSCs) that were obtained by successful reprogramming of mouse and human somatic cells after expression of a limited number of defined transcription factors [17-19].

The possibility to generate autologous pluripotent stem cells from adults could also reduce immunological problems that are expected to occur when allogeneic ESCs are used for regenerative therapies [20,21]. However, in the case of maGSC-based therapies only male patients could theoretically benefit from fully autologous stem cells. The complexity of maGSC generation and requirements for quality assurance for individual stem cell lines might be a further hurdle for the therapeutic use of individualized maGSCs, as has been discussed in the context of iPSCs [22,23]. Thus, although maGSCs and iPSC could theoretically allow the generation of autologous pluripotent stem cells, they might also be of use for therapeutic purposes in partially allogeneic settings. However, even when autologous stem cells are utilized for transplantation therapies, cytotoxic T lymphocytes (CTLs) might recognize "oncofetal" antigens [24] expressed by pluripotent stem cells or differentiation antigens [25] as has been documented and exploited in the context of tumor immunology. A further concern is that syngeneic or autologous transplantations might be associated with a higher risk of teratoma growth than allogeneic transplantations if grafts are derived from pluripotent cells and are contaminated with undifferentiated cells [26]. Therefore, it seems important to determine whether new pluripotent stem cell types such as maGSCs and iPSCs are protected against CTLs, similarly to what has been reported for ESCs [27].

In this study, we have analyzed the teratoma growth of maGSCs depending on the conditions used for their culture and *in vitro *differentiation. Furthermore, the susceptibility of three maGSC lines (maGSC Stra8 SSC5, maGSC 129/Sv, maGSC C57BL) to CTLs has been determined in comparison to corresponding ESC lines (ESC Stra8, ESC 129/Sv R1, ESC C57BL), iPSCs, and F9 teratocarcinoma cells.

## Methods

### Cell culture

The maGSC line Stra8 SSC5 (H2^b^) [3] was grown under four different culture conditions that have been previously described in detail [7]. Briefly, under condition I the cells were cultured in dishes, pre-coated with 0.1% gelatine (Sigma-Aldrich, Taufkirchen, Germany) for at least 1 h at 4°C, with basic medium, i.e. Dulbecco's modified Eagle's medium (DMEM) supplemented with 15% fetal calf serum (FCS, selected batches), 2 mM L-glutamine (Invitrogen, Karlsruhe, Germany), 50 μM β-mercaptoethanol (β-ME; Promega, Mannheim, Germany), 1 × nonessential amino acids (NEAA; Invitrogen). For condition II the cells were cultured in gelatine-coated dishes with basic medium containing 10^3 ^units/ml leukaemia inhibitory factor (LIF; ESGRO, Millipore, Billerica, MA, USA). Under condition III the cells were cultured on a feeder layer of mitomycin C-inactivated mouse embryonic fibroblasts (MEFs) with basic medium. Cells under condition IV were cultured on a mitomycin C-inactivated MEF feeder layer with basic medium containing 10^3 ^units/ml LIF. The cells were used at passages 12 to 18 (condition I), 9 to 20 (condition II), 8 to 22 (condition III), and 10 to 22 (condition IV). The other pluripotent stem cell lines used in this study were cultured under condition IV. These included the lines maGSC 129/Sv (H2^b^) [3] (passages 24 to 37), maGSC C57BL (H2^b^) [3] (passages 24 to 31), ESC 129/Sv MPI-II (H2^b^) [28] (passages 24 to 33), and ESC 129/Sv R1 (H2^b^) [29] (passages 19 to 38). The line ESC Stra8 SSC5 (passages 10 to 15) was derived from a Stra8-EGFP/Rosa26 transgenic mouse [8]. A new ESC line (ESC C57BL) (H2^b^) (passages 9 to 18) was generated as described [30]. The iPSCs (passages 13 to 21) were derived from MEFs (129/Sv × C57BL/6 F1) (H2^b^) and have been described previously [31]. They were kindly provided by Dr. Rudolf Jaenisch, The Whitehead Institute for Biomedical Research, Cambridge, USA. F9 teratocarcinoma cells (H2^b^), which originated from an embryonal testicular teratocarcinoma of a 129/Sv mouse were cultured as described [32]. The mouse cell lines YAC-1 (H2^a^), RMA (H2^b^), and RMA-S (H2^b^) were maintained in NaHCO_3_-buffered DMEM supplemented with 10% FCS (Biochrom, Berlin, Germany), 2 mM L-glutamine, 1 mM sodium pyruvate, 50 μM β-ME, 100 U/ml penicillin, and 100 μg/ml streptomycin.

### In vitro differentiation of maGSCs

For differentiation, maGSC Stra8 SSC5 cells cultured under condition III were cultivated as embryoid bodies (EBs) in hanging drops as previously described [3]. Briefly, maGSC Stra8 SSC5 cells were cultured without MEFs for 2 passages before cultivation as EBs. 400 cells in 20 μl of differentiation medium (Iscove's modified Dulbecco's medium supplemented with 20% FCS, 2 mM L-glutamine, 1× NEAA, and 450 μM α-monothioglycerol 3-mercapto-1,2-propandiol (MTG; Sigma-Aldrich, Taufkirchen, Germany)) were placed on the lids of Petri dishes filled with phosphate-buffered saline (PBS) and incubated in hanging drops for 2 days and in bacteriological Petri dishes for another 3 days. At day 5, single EBs were transferred into gelatine (0.1%)-coated 6-cm tissue culture dishes in differentiation medium for 10 further days.

### Generation of CTLs and ^51^Chromium release assays

To obtain peptide-specific CTLs, spleen cells from naive OT-I mice [33] were stimulated *in vitro *in the presence of 1 nM SIINFEKL peptide (Ovalbumin 257-264; Bachem Biochemica, Heidelberg, Germany) as previously described [34]. Target cells were labeled by incubating 1 × 10^6 ^cells in 200 μl N-2-hydroxyethylpiperazine-N'-2-ethanesulfonic acid (HEPES)-buffered DMEM containing 100 μl FCS and 50 μCi Na_2_^51^CrO_4 _(Hartmann Analytic, Braunschweig, Germany) for 1 h at 37°C and washed three times with HEPES-buffered DMEM. Effector cells were added to 5 × 10^3 51^Cr-labeled target cells in triplicate at various ratios in 200 μl HEPES-buffered DMEM/10% FCS per well of round-bottomed microtiter plates. Spontaneous release was determined by incubation of target cells in the absence of CTL. To allow for a peptide-specific killing the respective wells were supplemented with 0.5 μg/ml SIINFEKL. To determine calcium-dependency of killing 2 mM ethyleneglycol-bis(b-aminoethyl ester)-N,N,N',N'-tetraacetic acid (EGTA) and 4 mM MgCl_2 _were added. The microtiter plates were centrifuged for 5 min at 40 × g, incubated at 37°C for 4 h, and then centrifuged again. Supernatant and sediment were separately taken to determine radioactivity in each well using a Wallac MicroBeta Trilux counter (PerkinElmer Life Sciences, Köln, Germany). Percentage of specific lysis was calculated by subtracting percent spontaneous ^51^Cr release [35].

### Flow cytometry

Flow cytometry was performed on a FACScan™ flow cytometer (BD Biosciences, Heidelberg, Germany) using CellQuest™ data acquisition and analysis software. The cell surface expression of major histocompatibility complex (MHC) class I molecules was analyzed using locus and haplotype-specific antibodies (Ab) anti-H2K^b ^(clone CTKb, mouse IgG_2a_, phycoerythrin (PE)-conjugated, Caltag Laboratories, Hamburg, Germany), and anti-H2D^b ^(clone CTDb, mouse IgG_2a_, PE-conjugated, Caltag). The isotype control (mouse IgG_2a_, PE-conjugated) was purchased from Caltag. To test whether the MHC class I expression can be stabilized at a lower temperature, the stem cells were incubated at 28°C for 24 h before analysis. In order to test for the stabilization of H2K^b ^cell surface expression by external peptides, the H2K^b^-restricted peptide SIINFEKL or a non-binding control peptide (NGLTLKNDFSRLEG) were added for 24 h to cell cultures. The expression analysis of pluripotency markers was done as described previously [3] using mouse anti-SSEA-1 (clone MC480, Developmental Studies Hybridoma Bank, IA, USA) and mouse anti-Oct4 (clone 9E3.2, Chemicon) mAbs.

### Gene expression analysis

Total RNA was extracted from cell lines using the TRIZOL^® ^reagent (Invitrogen, Carlsbad, USA) according to the manufacturer's instructions. The RNA was then treated with RNase free DNase (RQ1, Promega, Madison, USA) for 20 min at 37°C and purified by phenol-chloroform-isoamyl alcohol (25:24:1) extraction and precipitated with 1/10 volume 300 mM sodium acetate (pH 4.8) and 1 volume 2-propanol before washing with 70% ethanol and solving in RNase free water. The quantity of the extracted RNA was determined with a ND-1000 Spectrophotometer (NanoDrop Technologies, Wilmington, USA) and the quality was analyzed using a Bioanalyzer 2100 (Agilent Techonolgies, Santa Clara, CA, USA). For synthesis of cDNA random oligo primers (Promega, Madison, USA) were used. The reverse transcription of RNA was performed for 60 min at 37°C with M-MLV RT polymerase (Promega, Madison, USA) in a total volume of 25 μl. Gene expression levels were analyzed by quantitative real time polymerase chain reaction (qRT-PCR) assays using the following forward and reverse primers generated according to the indicated reference sequences: *H2K*^*b*^ (NM_001001892.2, 5'-CCT GGA GTG GAC TTG GTG AC-3' and 5'-GGT GTA GAG GGG TGG ACT GG-3') *H2D*^*b *^(NM_010380.3, 5'-CCC TGT GAG CTT GGG TTC AG-3' and 5'-ACA GGG CAG TGC AGG GAT AG-3'), transporter associated with antigen processing 1 (*Tap1*) (NM_001161730.1, 5'-CTG CTC TCC CTC TAC CCC TC-3' and 5'-CTG AGT GGA GAG CAA GGA GTC-3'), *Tap2 *(NM_011530.3, 5'-GCA GAC GAC TTC ATA GGG GA-3' and 5'-GTT GCT TCT GTC CCA CAG C-3'), β2-microglobulin (*B2m*) (NM_009735.3, 5'-CTC ACA CTG AAT TCA CCC CC-3' and 5'-CAG TAG ACG GTC TTG GGC TC-3'), and *Serpinb9 *(NM_011452.2, 5'-TGC AGA CAA AAC TTG TGA AGT CCT C-3' and 5'-TGC CTG GAC ACC TCT GCT TC-3') encoding the serine protease inhibitor 6 (SPI-6) protein. The mRNA expression of the housekeeping gene hypoxanthine guanine phosphoribosyl transferase 1 (*Hprt1*) (NM_013556.2, 5'-GTC CTG TGG CCA TCT GCC TA-3' and 5'-GGG ACG CAG CAA CTG ACA TT-3') was always monitored as internal control. Amplification reactions were carried out in 96-well plates in 25 μl reaction volumes with the Power SYBR^® ^green PCR master mix (Applied Biosystems, Foster City, USA). The PCR reaction plates were preheated for 2 min at 50°C and for 10 min at 95°C followed by 40 cycles of denaturation (15 s at 95°C) and amplification (1 min at 60°C). All reactions were performed in technical triplicates using an ABI 7500 Real Time PCR System. For the data analysis, the ABI 7500 SDS software (Applied Biosystems) was used. The variations in cDNA concentration in different samples were normalized to the housekeeping gene *Hprt1*. The cycle threshold (ct) values obtained for the genes of interest were corrected by the ct value obtained for *Hprt1 *in the same sample. The relative level of transcripts was then expressed as Δct value (ct for *Hprt1 *minus ct for the gene of interest).

### Immunoblot

Proteins from cultured cells were isolated as described previously [8]. The protein extracts (20 μg per lane) were separated by sodiumdodecyl sulphate-polyacrylamide gel electrophoresis (SDS-PAGE) and transferred to nitrocellulose (Schleicher und Schüll, Dassel, Germany). The membranes were stained with an anti-PI-9 mAb (clone JM-3544, mouse IgG, MBL, Woburn, MA, USA) that has been described to cross-react with its mouse homologue SPI-6 [27]. An anti-heat shock cognate 70 (HSC70 or HSPA8) mAb (clone 1B5, rat IgG2a, StressGen, Biomol, Hamburg, Germany) was used as loading control. The primary antibodies were diluted 1:2000 in PBS/0.05% Tween 20. Subsequently, blots were incubated with secondary goat anti-mouse IgG (115-005-003, Jackson Laboratories, Dianova, Hamburg, Germany) and goat anti-rat IgG and IgM (112-005-068, Jackson Laboratories) Abs and then with a tertiary peroxidase-conjugated rabbit anti-goat IgG Ab (305-035-045, Jackson Laboratories, Dianova) at dilutions of 1:5000. The substrate reaction was carried out with 0.05% 3,3'-diaminobenzidine/0.0003% H_2_O_2 _in PBS/0.05% Tween 20.

### Animal experiments

All animals were bred in the central animal facility of the Medical Faculty of the University of Göttingen. Severe combined immunodeficient SCID/beige mice (C.B-17/IcrHsd-scid-bg) were kept under pathogen-free conditions. All animal experiments had been approved by the local government. For the analysis of subcutaneous tumor growth undifferentiated maGSC and *in vitro *differentiated cells, respectively, were suspended in 100 μl PBS (2 × 10^6 ^cells/100 μl) and injected into the flank of the animals. Tumor growth was monitored every second day by palpation and size was recorded using linear calipers. The tumor volume was calculated by the formula V = πabc/2, where a, b, c are the orthogonal diameters. Animals were sacrificed at day 100, or before when a tumor volume of 1 cm^3 ^was reached, when a weight loss of more than 10% occurred, or when any behavioral signs of pain or suffering became evident. Autopsies were performed for all animals. Tumor tissue was immediately frozen in liquid nitrogen or placed in phosphate-buffered 4% formalin for 16 h and then embedded in paraffin. Tissue sections (2 μm) were stained with hematoxylin and eosin (HE) for histological examinations.

### Statistics

The data were analyzed using the non-parametric Kruskal Wallis test and the U test for subgroup analysis with the WinSTAT software. A significance level of α = 0.05 was used.

## Results

### Maintenance of maGSC Stra8 SSC5 cells under different culture conditions

**Figure 1:**
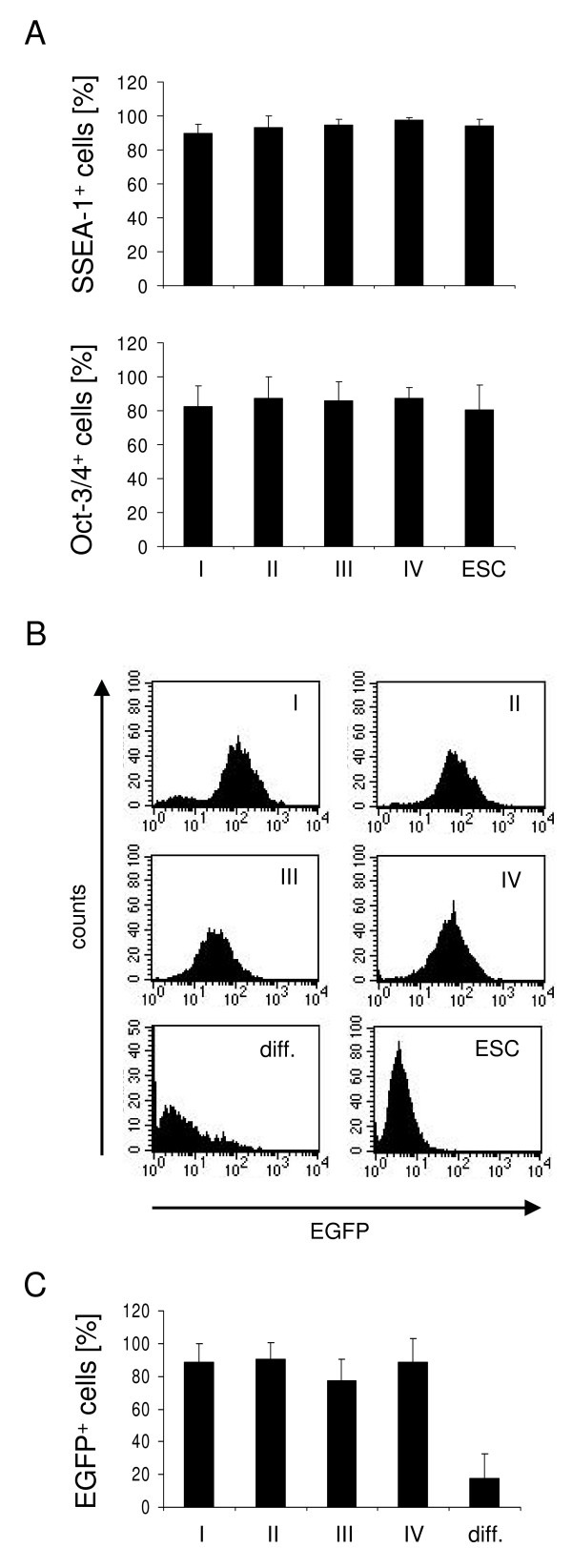
**Proportion of pluripotent cells among maGSC Stra8 SSC5 cells cultured under various conditions**. (A) MaGSC Stra8 SSC5 cells were cultured in basic medium (condition I), medium plus LIF (condition II), basic medium on MEFs (condition III), in medium plus LIF on MEFs (condition IV). ESCs (line ESC 129Sv MPI-II) were cultured under condition IV. The proportion of SSEA-1 and Oct-3/4-positive cells was evaluated by flow cytometry. The means of EGFP-positive cells + standard deviation (SD) are shown (n = 3). (B) MaGSC Stra8 SSC5 cells cultured under conditions I to IV or cells differentiated *in vitro *for 5 plus 10 days (diff.) were analyzed for EGFP expression by flow cytometry. Representative histograms are shown. An EGFP-negative ESC line (ESC 129Sv MPI-II) is included as negative control. (C) The means of EGFP-positive cells + SD are shown (n = 5).

MaGSC lines can be grown *in vitro *under different cell culture conditions [3,7], of which we used four in our study. Condition I consists of basic medium, condition II is the basic medium plus LIF, condition III is the basic medium plus a feeder layer of MEFs, and condition IV is the basic medium plus LIF plus MEFs. The condition IV represents the gold standard for ESC culture. The maGSC line SSC5 expressed the pluripotency markers stage-specific embryonic antigen 1 (SSEA-1) and Oct3/4 under all culture conditions similarly to the ESC line MPI-II (Figure 1A). This maGSC line was generated from the Stra8-EGFP/Rosa26 transgenic mouse, which expresses the enhanced green fluorescent protein (EGFP) under control of the *Stra8 *promoter [3]. In male mice the *Stra8 *promoter is selectively active in spermatogonia and their immediate descendants (preleptotene spermatocytes) [36-39] before it becomes silenced upon further differentiation. Therefore, the undifferentiated maGSC Stra8 SSC5 cells are characterized by EGFP expression [3]. We determined the proportion of EGFP expressing cells under the four culture conditions and after *in vitro *differentiation for 5 plus 10 days by flow cytometry as exemplified in Figure 1B. Under all four culture conditions more than 75% of the maGSC Stra8 SSC5 cells expressed EGFP (Figure 1C) and the conditions did not differ significantly (p = 0.1753, Kruskal-Wallis test). Thus, maGSCs retain expression of pluripotency markers even under culture conditions without either LIF or feeder cells, at least for more than 16 passages. After spontaneous *in vitro *differentiation using the hanging drop method, on average less than 20% of the cells remained positive for EGFP at day 10 after plating EBs at day 5 (Figure 1C).

### Teratoma formation of maGSC Stra8 SSC5 cells cultured under different culture conditions

**Figure 2:**
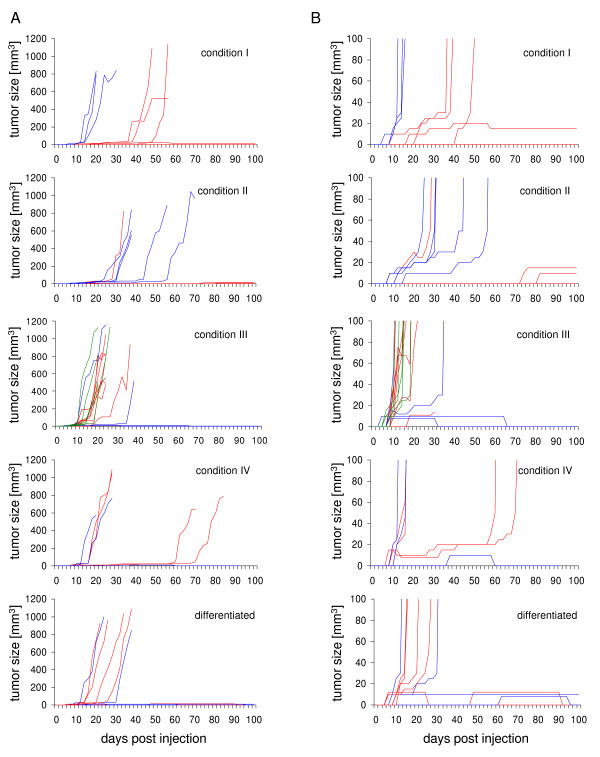
**Tumor growth of maGSC Stra8 SSC5 cells after injection into immunodeficient SCID/beige mice**. Undifferentiated maGSC Stra8 SSC5 cells or *in vitro *differentiated cells (5 + 10 days) were injected subcutaneously at day 0 into SCID/beige mice (2 × 10^6 ^cells per mouse). The undifferentiated cells were cultured under the four conditions (I to IV) before the injection. The tumor size was recorded every second day until day 100 using linear calipers. (A) The growth of tumors in individual mice is shown. The mice were injected with cells from two to three independent cultures that are indicated by different colors. Animals were sacrificed after day 100 or before when ethical criteria for the stop of the experiment were reached. (B) The same data are presented on a different scale for the tumor size in order to show small or regressing tumors.

**Figure 3:**
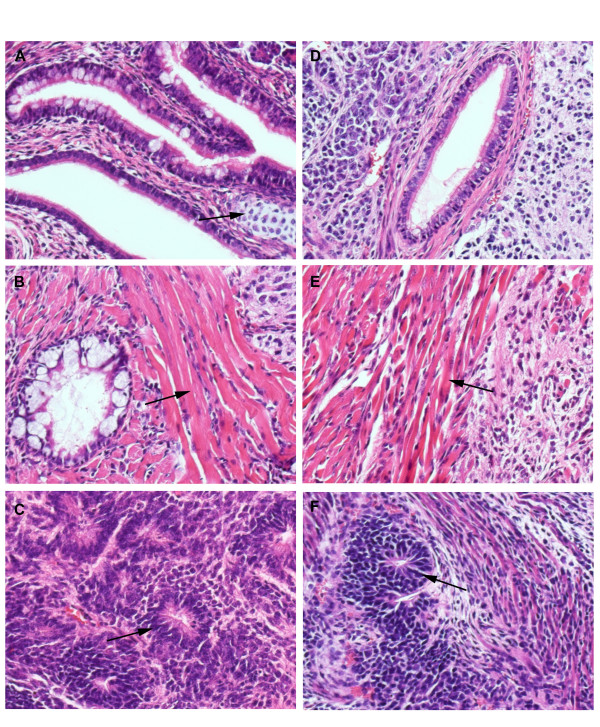
**Histology of teratomas formed after injection of maGSC Stra8 SSC5 cells and differentiated cells into immunodeficient SCID/beige mice**. (A-C) Histological analysis of a representative teratoma derived from maGSC Stra8 SSC5 cells under condition I. (D-F) Histology of a teratoma derived from *in vitro *differentiated cells. The formed teratomas contained derivatives of all three embryonic germ layers (ectoderm, mesoderm, and endoderm). (A, D) Gut epithelium (endoderm). (A) Cartilage at arrow (mesoderm). (B, E) A rivulet of muscle at arrow (mesoderm). (C, F) Neural-like tissues at arrow (ectoderm). All images were obtained from formalin-fixed and paraffin-embedded teratoma sections stained with hematoxylin and eosin.

We have previously shown that maGSC Stra8 SSC5 cells cultured in basic medium on a feeder layer (condition III) have the potential to form teratomas in immunodeficient SCID/beige mice [3]. To determine whether the various cell culture conditions have an effect on the pluripotency of maGSCs, we compared the tumor growth of maGSC Stra8 SSC5 cells cultured under the conditions I to IV after subcutaneous injection into SCID/beige mice. The passage numbers of maGSC Stra8 SCC5 cells used for these experiments was between 12 and 22. Tumors were found in high frequency within 100 days after injection of maGSC Stra8 SSC5 cells cultured under all conditions (Table 1). In most cases, progressively growing tumors occurred within the first 50 days (Figure 2). However, some tumors remained small and stable for the whole observation time (Figure 2); others even regressed before the end of the experiment (Figure 2, Table 1). Usually, the tumors were found only subcutaneously at the site of injection. In a few cases, autopsy revealed that tumors had grown invasively and given rise to tumors in the peritoneal cavity (Table 1). The histopathological evaluation of tumors indicated that teratomas had formed from the maGSC Stra8 SSC5 cells cultured under four different conditions. This is exemplified in Figures 3A-C representing a teratoma derived from maGSC Stra8 SSC5 cells cultured under condition I. The teratomas contained derivatives of all three embryonic germ layers, including epithelium with intestinal differentiation (endoderm, Figure 3A), striated muscle (Figure 3B), smooth muscle, fat, bone and cartilage (mesoderm), and neural tissue (ectoderm, Figure 3C). Thus, the cells cultured under all four conditions were undifferentiated, as indicated by EGFP expression and expression of pluripotency markers SSEA-1 and Oct3/4 (Figure 1), and also pluripotent as shown by the capability to form teratomas.

**Table 1:** Tumor formation of maGSC Stra 8 SSC5 cells in immunodeficient SCID/beige mice

**Culture condition**	**Tumor frequency**	**Regression frequency**	**Invasive growth**
I	88% (7/8)	0% (0/7)	29% (2/7)

II	80% (8/10)	0% (0/8)	13% (1/8)

III	100% (18/18)	11% (2/18)	6% (1/18)

IV	100% (7/7)	14% (1/7)	14% (1/7)

diff.	83% (11/12)	27% (3/11)	0% (0/11)

MaGSC Stra8 SSC5 cells cultured under various conditions (I to IV) and cells differentiated *in vitro *for 5 plus 10 days (diff.) were injected subcutaneously into the flank of SCID/beige mice (2 × 10^6 ^cells in PBS/animal). The percentage and number of animals in which tumors were found during autopsy or in which tumors were palpable (at least during 3 consecutive observations) at the side of injection before day 100 after injection is indicated. In addition, the percentages and numbers of animals are given in which tumors were present but regressed before day 100 and in which an invasive growth was observed. The differentiated cells (diff.) were obtained as described in the Materials and Methods section from maGSC Stra8 SSC5 cells that had been cultured under condition III.

In addition to the undifferentiated cells, *in vitro *pre-differentiated cells (5 + 10 days of differentiation culture) were injected into 12 SCID/beige mice. In 6 cases an early progressive tumor growth was observed, 2 tumors remained small and stable, and 3 regressed before day 100 (Figure 2, Table 1). Thus, the *in vitro *pre-differentiation protocol used here is not sufficient to deplete tumorigenic cells from the cultures. This finding is in agreement with the observation that a significant proportion of the *in vitro *differentiated cells still expressed EGFP, indicating the presence of undifferentiated cells (Figure 1B, C). The histopathological analysis of these tumors showed that the *in vitro *differentiated cells, similar to undifferentiated maGSCs, formed teratomas (Figure 3D-F). The teratomas contained derivatives of three embryonic germ layers, including epithelium with intestinal differentiation (endoderm, Figure 3D), striated muscle (mesoderm; Figure 3E), smooth muscle, fat, bone and cartilage (mesoderm), and neural tissue (ectoderm, Figure 3F).

The maGSC Stra8 SSC5 cells cultured under condition III (on feeder cells) were also injected subcutaneously into immunocompetent C57BL/6 mice. None of 9 animals receiving 2 × 10^6 ^cells developed a teratoma within 100 days. At autopsy no residues of the injected cells were found (data not shown). Thus, maGSCs were readily rejected in immunocompetent allogeneic hosts.

### Expression of MHC class I molecules on maGSC Stra8 SSC5 cells cultured under various conditions

**Figure 4:**
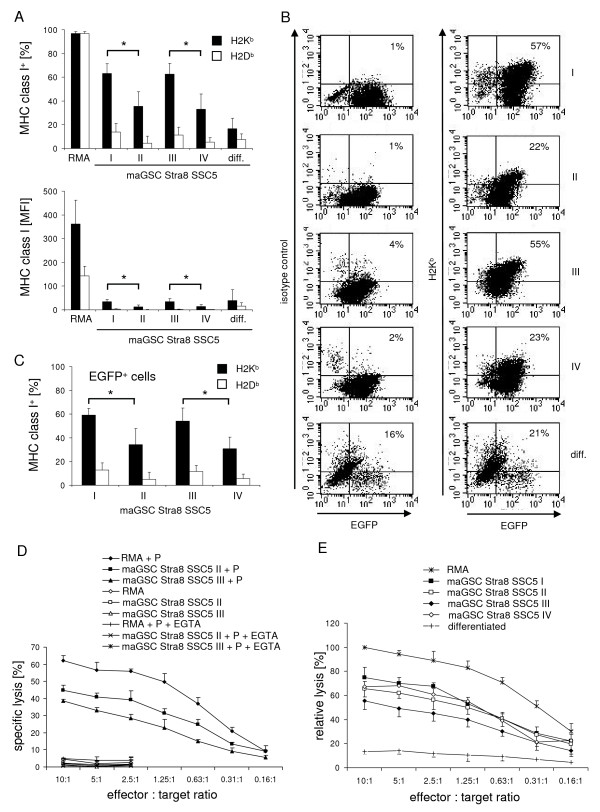
**MaGSC Stra8 SSC5 cells can be killed by CTLs despite low expression of MHC class I molecules**. (A) MaGSC Stra8 SSC5 cells cultured under conditions I, II, III, and IV, *in vitro *differentiated maGSC Stra8 SSC5 cells (diff.), and RMA cells were analyzed for MHC class I expression (H2K^b ^and H2D^b^) by flow cytometry. The mean percentages of positive cells + SD (staining with MHC class I mAbs minus isotype control) and the mean fluorescence intensities (MFI) + SD are given as determined in 5 experiments. Significant differences between culture conditions that differed in the presence of LIF are indicated (* p < 0.05, U test). (B) Individual dot plots from these experiments are shown. The left panel shows staining with an isotype control and the right panel with an anti-H2K^b ^mAb. The percentages of EGFP and H2K^b ^(or isotype control) double positive cells are indicated in the upper right quads. (C) The evaluation of the proportion of MHC class I-positive cells in the experiments was restricted to the EGFP-positive maGSC Stra8 SSC5 cell population. Significant differences between culture conditions that differed in the presence of LIF are indicated (* p = 0.05, U test). (D) A representative experiment is shown in which the susceptibility of maGSC Stra8 SSC5 cells cultured under conditions II and III to CTLs was determined. RMA cells served as highly CTL susceptible controls. The target cells were pulsed with the SIINFEKL peptide P (0.5 μg/ml) and exposed to CTLs derived from TCR-transgenic OT-I mice. The mean of specific lysis plus SD at different effector:target ratios (10:1 to 0.16:1) in the presence or absence of the SIINFEKL peptide measured in a ^51^Cr release assay is shown. To confirm granule exocytosis dependency of killing EGTA was added to the test. (E) The means of relative lysis ± SD of the various target cell lines by OT-I CTLs are shown as determined in three independent experiments. The mean percentage of specific lysis of RMA cells (serving as positive control) at the highest effector to target ratio (10:1) was adjusted to 100% in each test and the relative lysis of the various target cells at different effector to target ratios was calculated.

Mechanisms of the immune system play an important role for teratoma growth in immunocompetent animals. To analyze the immunogenicity of maGSC Stra8 SSC5 cells, we determined the expression of MHC class I molecules and their susceptibility to CTL-mediated killing. A proportion of the maGSC Stra8 SSC5 cells expressed MHC class I molecules (Figure 4A, B). Between 35 and 60% of the cells cultured under the four different conditions expressed H2K^b ^molecules and less than 20% of the cells expressed H2D^b ^molecules as detected by flow cytometry. The proportion of H2K^b ^expressing cells differed between the cells cultured under the four conditions (p = 0.0127, Kruskal-Wallis test) and was higher in cultures without LIF (conditions I and III) compared to the respective condition with LIF suggesting an effect of the culture conditions on the immunologic phenotype of the maGSC Stra8 SSC5 cells. The results were similar (p = 0.0126, Kruskal-Wallis test) when the analysis was restricted to the undifferentiated Stra8-EGFP expressing cells (Figure 4C). The expression levels as determined by the mean fluorescence intensity (MFI) on maGSC Stra8 SSC5 cells was rather low (on average <50) and much lower than on RMA cells (on average >350) that were used as positive control for the staining (Figure 4A). However, the MFI for H2K^b ^also differed between the conditions (p = 0.0151, Kruskal-Wallis test) and was higher in cultures without LIF. The *in vitro *differentiation of SSC5 cells reduced the proportion of H2K^b ^expressing cells (Figure 4A).

### MaGSC Stra8 SSC5 cells cultured under different conditions are killed by CTLs whereas in vitro differentiated cells acquire resistance

Next we tested whether the maGSC Stra8 SSC5 cells can be killed by CTLs. We used CTLs from TCR-transgenic OT-I mice as effector cells, which recognize the ovalbumin-derived peptide SIINFEKL in an H2K^b^-restricted manner [33]. The target cells, which do not express ovalbumin, were pulsed with the peptide to allow for binding to H2K^b ^molecules and peptide-dependent killing. RMA cells served as positive control. The results of a representative individual experiment are shown in Figure 4D. RMA cells as well as maGSC Stra8 SSC5 cells were not killed when they were not pulsed with the peptide. They were readily killed in an effector dose-dependent manner when the SIINFEKL peptide was present. In addition, the killing could be blocked by EGTA indicating that the lysis of the targets was mediated by the calcium-dependent granule exocytosis pathway. A summary of the results of 3 experiments with maGSC Stra8 SSC5 target cells cultured under the four conditions and differentiated cells, respectively, is shown in Figure 4E. The lysis of the maGSC Stra8 SSC5 targets did not directly correlate with the H2K^b ^expression levels since the maGSC Stra8 SSC5 cells cultured under condition III showed the highest expression levels of H2K^b ^but the lowest killing in comparison to the other three culture conditions. Interestingly, the *in vitro *differentiated cells (5 + 10 days of differentiation culture) were hardly killed by CTLs.

### Further pluripotent cell lines are killed by CTLs despite being negative for MHC class I expression in flow cytometry

**Figure 5:**
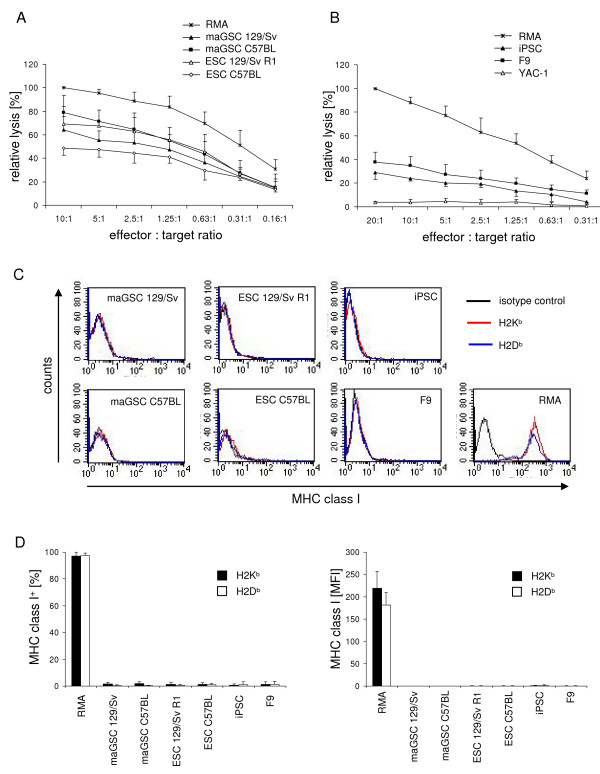
**Pluripotent cells can be killed by CTLs despite very low expression of MHC class I molecules**. (A) The means of relative lysis ± SD of maGSC and ESC lines by CTLs are shown as determined in three independent experiments. The target cells were pulsed with the SIINFEKL peptide (0.5 μg/ml) and exposed to CTLs derived from TCR-transgenic OT-I mice. For normalization between experiments, the mean percentage of specific lysis of RMA cells (serving as positive control) at the highest effector to target ratio (10:1) was adjusted to 100% in each test and the relative lysis of the various target cells at different effector to target ratios was calculated. (B) The means of relative lysis ± SD of iPSCs and F9 teratocarcinoma cells by CTLs are shown as determined in three independent experiments. The target cells were pulsed with the SIINFEKL peptide (0.5 μg/ml) and exposed to CTLs derived from TCR-transgenic OT-I mice. The relative lysis was calculated as described above. YAC-1 cells (H2^a^) served as a negative control. (C) The target cells were tested in each experiment for MHC class I expression (H2K^b ^and H2D^b^) by flow cytometry. Representative individual histograms are shown. (D) The mean percentages of positive cells + SD (staining with MHC class I mAbs minus isotype control) and the MFI + SD are given for the three experiments.

We then analyzed the killing of two further maGSC lines of the haplotype H2^b ^(maGSC 129/Sv and maGSC C57BL) by CTL and compared them to ESC targets (ESC 129/Sv R1, and ESC C57BL). These cells were cultured in the presence of LIF on feeder cells (condition IV) and transferred two days before the experiment to gelatine-coated dishes (condition II). A summary of the results of 3 experiments is shown in Figure 5A. Similar to the maGSC Stra8 SSC5 line, both maGSC lines (maGSC 129/Sv and maGSC C57BL) were highly susceptible to CTL killing. The relative lysis was between 60 and 80% compared to RMA cells. The lysis of the ESC line 129/Sv R1 was in a similar range. The ESC line C57BL was killed but less efficiently. The relative lysis was between 40 and 50% compared to RMA cells. The killing of all target cells was completely peptide-dependent (data not shown). Next we used iPSCs and F9 teratocarcinoma cells as CTL targets. A summary of the results of 3 experiments is shown in Figure 5B. Both cell types were killed in a peptide-dependent manner although less efficiently than RMA cells. The relative lysis was between 20 and 40% compared to RMA cells. YAC-1 cells (H2^a^) which are a target for natural killer (NK) cells were not killed, further confirming the specificity of the CTL-mediated killing and the absence of NK cell-mediated killing.

In parallel to the killing assays, the MHC class I expression on the target cells was determined by flow cytometry. Interestingly, the maGSC lines maGSC 129/Sv and maGSC C57BL, in contrast to the maGSC Stra8 SSC5 cell line, did not express MHC class I molecules at a level detectable by flow cytometry (Figure 5C, D). They were very similar to the MHC class I negative ESC lines 129/Sv R1 and C57BL (Figure 5C, D). In addition, also the iPSCs and F9 teratocarcinoma cells were negative for MHC class I molecules in flow cytometry (Figure 5C, D). The external peptide pulsing of targets was apparently so efficient that even maGSCs, ESCs, iPSC and F9 cells, on which no H2K^b ^molecules could be detected by flow cytometry, were readily killed in a peptide-specific manner.

### The expression of MHC class I peptides on maGSCs and ESCs cannot be rescued by decreased temperature or external peptides

**Figure 6:**
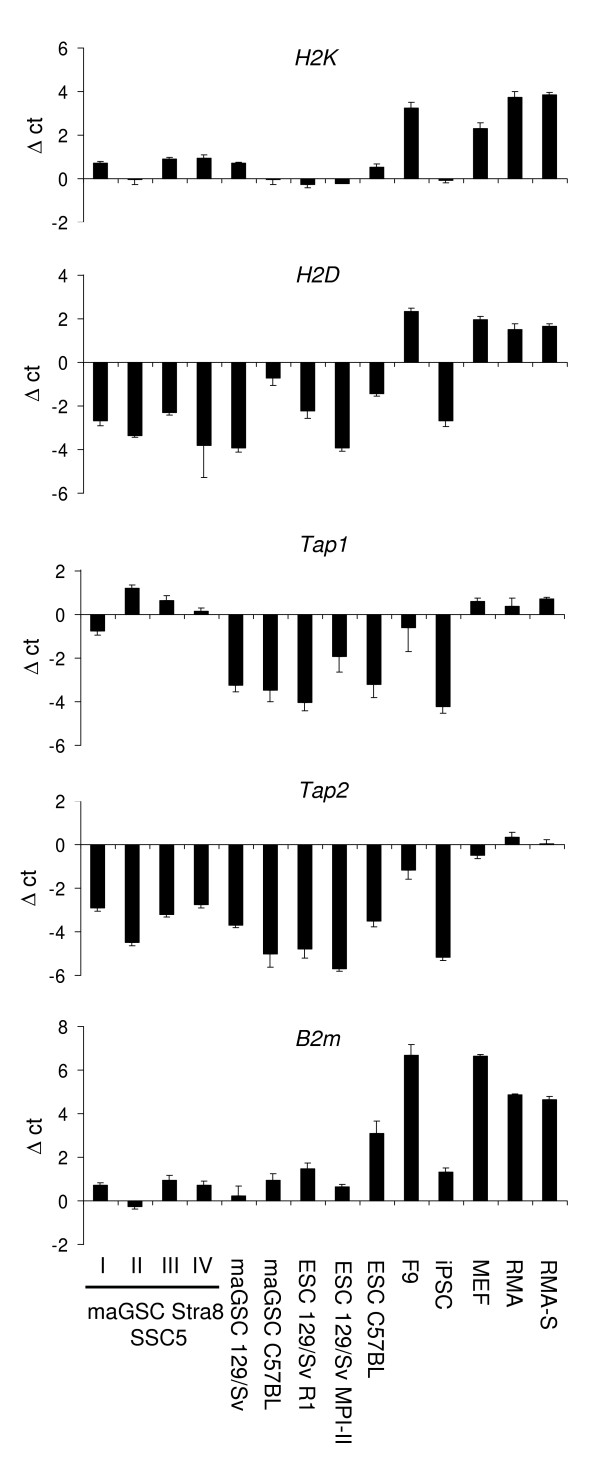
**Quantitative real time PCR analysis of *H2K*, *H2D*, *Tap1*, *Tap2*, and *B2m *expression in stem cell lines**. The transcripts of *H2K*, *H2D*, *Tap1*, *Tap2*, and *B2m *genes were analyzed in triplicates by qRT-PCR in the indicated cell lines in comparison to the housekeeping gene *Hprt1*. The maGSC Stra8 SSC5 cells had been cultured under conditions I, II, III, and IV before mRNA preparation. Means and SDs of the Δct values (*Hprt *minus gene of interest) are shown.

The pluripotent stem cells could either completely fail to express MHC class I molecules or have very few MHC molecules at the cell surface due to a deficiency in peptide loading, as e.g. neuronal cells that express low levels of TAP molecules [40] or RMA-S cells that have a mutation in the *Tap2 *gene [41,42]. We analyzed the expression of *H2K*, *H2D*, *Tap1*, *Tap2*, and β2-microglobulin (*B2m*) at the mRNA level by qRT-PCR (Figure 6). The pluripotent stem cell lines expressed *H2K *and *B2m *mRNA although at lower levels than RMA and RMA-S cells. The *H2D *mRNA was less expressed in the stem cell lines. The expression of *Tap1 *and *Tap2 *was indeed low in most stem cell lines. Only maGSC Stra8 SSC cells that expressed H2K^b ^molecules at the cell surface (Figure 4A, B) had similar *Tap1 *mRNA levels as RMA cells. F9 teratocarcinoma cells were the only pluripotent cell type that expressed all these mRNAs at rather high levels. Interestingly, iPSCs expressed much less of these mRNAs than MEFs that were used for their generation.

**Figure 7:**
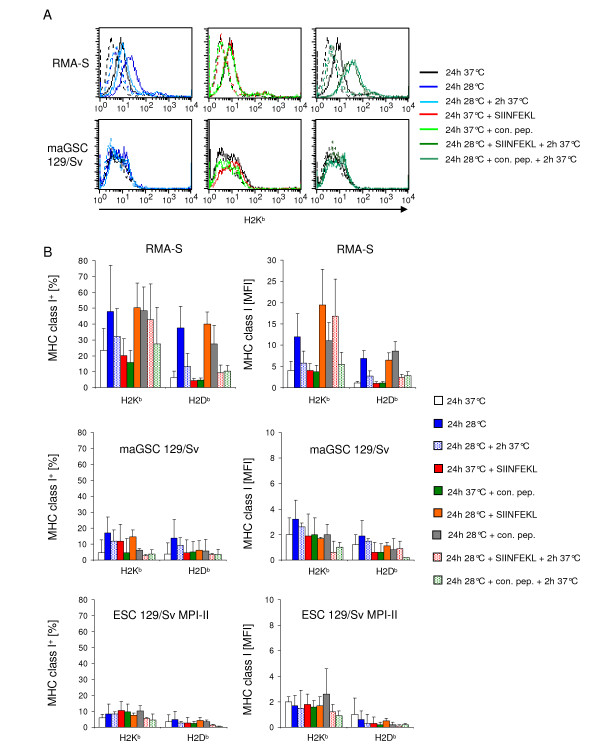
**Analysis of MHC class I expression on stem cell lines at low temperature and in the presence of peptides**. MaGSC 129/Sv and ESC 129/Sv MPI-II cells and as positive control RMA-S cells were cultured for 24 h at 37°C or at 28°C, in the presence of the H2K^b^-binding peptide SIINFEKL (0.5 μg/ml) or a non-binding control peptide (con. pep.) NGLTLKNDFSRLEG. The culture at 28°C was either followed by a further incubation at 37°C for 2 h or not before flow cytometric analysis of H2K^b ^and H2D^b ^cell surface expression. (A) An individual experiment with RMA-S and maGSC 129/Sv cell is shown by histogram overlays. The different experimental conditions are indicated by the colors of the lines. The H2K^b ^staining is indicated by full lines and staining with the isotype control by dotted lines. (B) The mean percentages of three independent experiments for H2K^b ^and H2D^b ^positive cells + SD (staining with MHC class I mAbs minus isotype control) and the MFI + SD are given.

The mRNA expression data are in accordance with the hypothesis that the peptide loading might be impaired in the pluripotent stem cell lines. Thus, we tested the expression of MHC class I molecules at decreased temperature and in the presence of external peptides. Incubation at 28°C for 24 h stabilized unloaded MHC class I molecules on RMA-S cells (Figures 7A, B), whereas a subsequent culture at normal temperature (2 h 37°C) decreased the cell surface expression again. A culture for 24 h at 37°C in the presence of the SIINFEKL peptide (0.5 μg/ml) did not increase the H2K^b ^expression as compared to untreated cells or cells that were incubated with a peptide that cannot bind to H2K^b ^(Figures 7A, B). The combination of culture at 28°C and presence of the SIINFEKL peptide further augmented the H2K^b ^but not H2D^b ^expression and the peptide-loaded H2K^b ^molecules were found to be more stable when the cells were transferred afterwards for 2 h to 37°C (Figures 7A, B). However, on the stem cell lines maGSC 129Sv and ESC 129Sv/MPI-II that were tested in parallel, MHC class I molecules were not similarly stabilized by these treatments. Thus, a low expression of TAP molecules is unlikely to be the main reason for the low MHC class I cell surface expression on these cell lines.

### MaGSCs as well as in vitro differentiated cells do not express the serine protease inhibitor 6 that can protect target cells against CTLs

**Figure 8:**
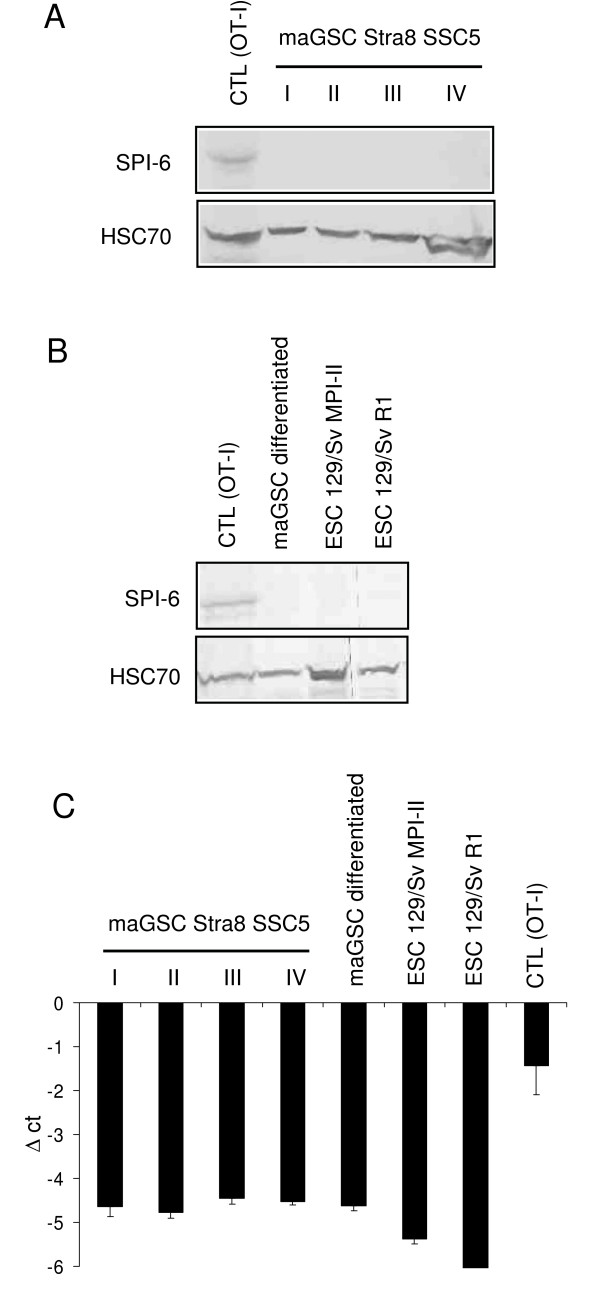
**Analysis of SPI-6 expression in stem cell lines**. (A) Protein extracts of maGSC Stra8 SSC5 cells cultured in basic medium (condition I), medium plus LIF (condition II), basic medium on MEFs (condition III), or in medium plus LIF on MEFs (condition IV) were separated by SDS-PAGE and the blot was probed with an anti-SPI-6 Ab and with an anti-HSC70 mAb as loading control. A protein extract of activated CTLs from OT-I mice served as positive control for SPI-6 detection. (B) The expression of SPI-6 was similarly probed in differentiated cells derived from maGSC Stra8 SSC5 cells and in two ESC lines for comparison. (C) Expression of *Serpinb9 *gene that encodes SPI-6, was analyzed in triplicates by qRT-PCR in the same cell lines in comparison to the housekeeping gene *Hprt1*. The maGSC Stra8 SSC5 cells had been cultured under conditions I, II, III, and IV before mRNA preparation. Means and SDs of the Δct values (*Hprt *minus gene of interest) are shown.

It has been reported that mouse ESCs express SPI-6 that confers protection against cellular cytotoxicity mediated by the granule exocytosis pathway [27,43]. We analyzed the expression of SPI-6 in maGSC Stra8 SSC5 cells cultured under the four conditions (I to IV) described above. The protein was expressed in activated CTLs from OT-I mice in which it contributes to the self protection against granzymes [44]. However, we did not detect the SPI-6 protein in the maGSCs (Figure 8A). In contrast to undifferentiated maGSCs, cells differentiated *in vitro *from maGSCs were rather resistant to lysis by CTLs (see Figure 4E). Therefore, we analyzed the expression of SPI-6 in cells differentiated from maGSC Stra8 SSC5 cells and included two ESC lines for comparison. The protein was neither detected in the *in vitro *differentiated cells nor in the ESC lines (Figure 8B). *Serpinb9 *that encodes SPI-6 was also hardly expressed in these cell lines at the mRNA level (Figure 8C). Similarly, we did not detect SPI-6 expression in any of the other pluripotent cell lines analyzed in this study (data not shown). Thus, expression of SPI-6 is apparently not responsible for the relative resistance of the *in vitro *differentiated maGSCs cells to CTLs that we observed.

## Discussion

One advantage of maGSCs over ESCs is that they could theoretically be a source of autologous pluripotent stem cells for individualized medicine. One criterion for assessing the pluripotency of stem cells is the capacity to give rise to teratomas in immunodeficient mice [45]. Therefore, the potential of maGSCs and their differentiation products to form teratomas has to be analyzed very carefully before any therapeutic application can be envisaged. In syngeneic or immunocompromised recipients, the risk of teratoma growth is inherent to transplantation therapies that are based on pluripotent cells. In immunocompetent allogeneic mice, transplanted ESCs do not usually form teratomas [26], unless very high numbers of pluripotent cells are injected [46]. We have compared the capacity of the maGSC line Stra8 SSC5 to give rise to teratomas depending on the culture conditions used to propagate them, and after differentiation. Cells cultured under four different conditions that varied in the presence of LIF and MEFs as feeder cells did not differ with respect to teratoma growth. This result was consistent with the presence of a similar proportion of undifferentiated cells under the four culture conditions as demonstrated by expression of pluripotency markers SSEA-1 and Oct-3/4, as well as EGFP expression driven by the *Stra8 *promoter in undifferentiated cells. Thus, the pluripotency of maGSCs was preserved under a variety of culture conditions for over 16 passages including those conditions that do not require feeder cells and might, therefore, have advantages in view of future therapeutic applications. Furthermore, cells differentiated *in vitro *(5 + 10 days of differentiation culture) without lineage selection still contained about 20% undifferentiated cells and gave rise to teratomas at high frequency in immunodeficient recipients, suggesting that lineage selection is necessary before the differentiated cells can be used for therapeutic application [47,48].

The risk of teratoma growth after transplantation of pluripotent cells does not only depend on the pluripotency of the injected cells but also on their immunogenicity and their susceptibility to cytotoxic effector mechanisms of the immune system because maGSC Stra8 SSC5 cells did not form teratomas in allogeneic immunocompetent mice. Therefore, we have started to characterize the immunological features of maGSCs and analyzed their susceptibility to killing by CTLs. CTLs recognize peptides presented by MHC class I molecules, which are highly polymorphic in the population. Alloreactive CTLs recognize MHC class I molecules either by direct or indirect allorecognition [20,21]. The most important immunological barrier for transplantation of pluripotent stem cell-derived allografts is presumably represented by MHC class I molecules [20,21], but minor histocompatibility antigens have also been shown to contribute to the rejection of ESC-derived grafts by CTL [49]. Interestingly, MHC class I molecules are not detectable by flow cytometry on mouse ESCs [26,27,50-54]. This is in contrast to human ESCs [55-57]. However, the expression of MHC class I molecules has been reported to increase during *in vitro *[55] or *in vivo *differentiation of mouse ESCs [54,58]. MHC class I molecules are inducible by interferon-γ in mouse ESCs [51,54] or in differentiated cells [27]. Thus, ESCs and their differentiation products might become targets for CTLs after transplantation [49,53,54,59].

Whilst the mouse maGSC line Stra8 SSC5 was found to express comparatively low levels of MHC class I molecules by flow cytometry, the other maGSC lines (maGSC 129/Sv and maGSC C57BL), ESCs, and iPSCs that were used for comparison were negative for these molecules, resembling the phenotype known for mouse ESCs and also F9 teratocarcinoma cells [60]. At the mRNA level *H2K *and *B2m *transcripts were detected in all pluripotent stem cell lines, although less abundantly than in RMA cells or MEFs. A low expression of *Tap1 *and *Tap2 *mRNA in most pluripotent stem cell lines suggested that a failure of peptide loading could explain the lack of MHC class I molecules at the plasma membrane. However, a decrease of temperature to 28°C or incubation with an H2K^b^-restricted peptide did not stabilize the MHC class I molecules on maGSC 129Sv or ECS 129Sv MPI-II cells. A previous report showed a marked increase of expression of H2D^d ^molecules on mouse embryonic cell lines (including one ESC line) that were transfected with an H2D^d^-expression construct and incubated with H2D^d^-binding peptides [61]. Thus, the endogenous *H2K *and *B2m *transcription in the stem cell lines analyzed here might still be insufficient to produce enough MHC class I molecules that can be detected by flow cytometry after stabilization by external peptides and decreased temperature.

However, all pluripotent cell lines analyzed here were killed by CTLs derived from TCR-transgenic OT-I mice that recognize the peptide SIINFEKL in an H2K^b^-restricted manner [33]. This finding is in accordance with studies showing that extremely low numbers of ligands on target cells are sufficient for CTL recognition and killing [62-64]. In addition, it has been reported previously that the MHC class I expression on mouse ESCs is sufficient for recognition by the SIINFEKL-specific T cell hybridoma B3Z [27,65]. We now show that the MHC class I expression level on ESCs, maGSCs, and iPSCs is functionally relevant since it is also sufficient for peptide-specific CTL killing, although the presence of the peptide did not markedly increase the H2K^b ^molecules at the cell surface of maGSCs and ESCs. The iPSCs appeared to be less susceptible to CTLs than the ESC and maGSC lines. Whether this is characteristic for iPSCs in general or only for the iPSC line analyzed here has to be determined. Interestingly, *in vitro *differentiation of maGSC Stra8 SSC5 cells appeared to reduce the sensitivity to CTLs because the *in vitro *differentiated target cells became much less sensitive to killing compared to their undifferentiated counterparts. The experiments using CTLs from TCR-transgenic mice demonstrated clearly that pluripotent stem cell lines can be killed by this mechanism. However, it has to be further clarified whether the antigen processing machinery of pluripotent stem cells is capable of generating peptide loaded MHC class I molecules for recognition by activated CTLs. Furthermore, it remains to be determined whether alloreactive CTLs that recognize their targets directly can kill the pluripotent stem cells with similar efficacy.

The observation that killing of the maGSCs, ESCs, iPSCs, and F9 cells could be inhibited by EGTA strongly suggests that the granule exocytosis pathway which involves perforin and granzymes [66,67] was used for killing. Recently, it has been reported that the mouse ESC line CGR8 is resistant to antigen-specific CTLs due to the expression of SPI-6 [27], which is an endogenous inhibitor of granzyme B [43]. We therefore looked for SPI-6 by western blot in the maGSC and ESC lines but could not detect expression of this protein in any of these cells, even after *in vitro *differentiation of maGSC Stra8 SSC5 cells. Their relative resistance to CTL cytotoxicity must therefore be caused by another mechanism. Our results are in accordance with recent reports by us [26] and others [65] showing that several ESC lines can be readily killed by NK cells. Although the mechanisms of target cell recognitions are different, NK cells and CTLs share the granule exocytosis pathway of target cell killing [66,67]. Thus, individual ESC lines might differ with respect to SPI-6 expression and resistance to CTL and possibly NK cells.

Several studies have suggested that mouse or human ESCs are immune-privileged. They may suppress immune responses by contact dependent [51,57] and contact independent mechanisms [46] or induce apoptosis in T cells [51]. It has also been reported that ESCs are resistant to CTL and NK cell-mediated cytotoxicity [27,46,51]. Here we have demonstrated that maGSC, ESC, and iPSC lines can be killed by activated peptide-specific CTLs despite levels of MHC class I expression that were below the detection level of flow cytometry. Therefore, it has to be assumed that these pluripotent cells can become targets for CTLs after transplantation.

## Conclusion

MaGSCs represent a new pluripotent cell type derived from adult mouse testis. They retain pluripotency under several cell culture conditions and form teratomas in immunodeficient mice. No expression of MHC class I molecules could be detected by flow cytometry in most maGSC lines and the iPSCs analyzed here, a phenotype shared with ESC lines and F9 teratocarcinoma cells. However, in immunocompetent recipients the immune response could suppress growth of the maGSCs lines as teratomas, and all analyzed pluripotent cells including maGSCs, ESCs, iPSCs, and F9 teratocarcinoma cells could be killed efficiently by activated CTLs. Thus, maGSCs, ESCs, and iPSCs can be recognized as targets for CTLs and they might be rejected by this mechanism after transplantation in immunocompetent recipients. However, the capacity of the immune system to kill pluripotent and teratoma forming stem cells could also be used to develop a new strategy to increase the safety of transplantation of *in vitro *differentiated cells. Indeed, it might be possible to selectively induce an adaptive immune response in recipients against antigens expressed selectively in pluripotent stem cells in order to kill any teratoma forming cells that could not be depleted before grafting. Further studies are required to validate or disprove this hypothesis.

## Abbreviations

Ab: antibody; B2m: β2-microglobulin; β-ME: β-mercaptoethanol; ct: cycle threshold; CTL; cytotoxic T lymphocyte; EB: embryoid bodies; EGFP: enhanced green fluorescent protein; EGTA: ethyleneglycol-bis(b-aminoethyl ester)-N,N,N',N'-tetraacetic acid; ESC: embryonic stem cell; DMEM: Dulbecco's modified Eagle's medium; FCS: fetal calf serum; HE: hematoxylin and eosin; HEPES: N-2-hydroxyethylpiperazine-N'-2-ethanesulfonic acid; HPRT1: hypoxanthine guanine phosphoribosyl transferase 1; HSC70: heat shock cognate 70; iPSC: induced pluripotent stem cell; LIF: leukemia inhibitory factor; mAb: monoclonal antibody; maGSC: multipotent adult germ-line stem cell; MEF: mouse embryonic fibroblast; MFI: mean fluorescence intensity; MHC: major histocompatibility complex; NEAA: nonessential amino acids; NK: natural killer; PBS: phosphate-buffered saline; qRT-PCR: quantitative real time polymerase chain reaction; SDS-PAGE: sodiumdodecyl sulphate-polyacrylamide gel electrophoresis; SPI-6: serine protease inhibitor 6; SSC: spermatogonial stem cell; SSEA-1: stage-specific embryonic antigen 1; TAP: transporter associated with antigen processing; TCR: T cell receptor

## Competing interests

The authors declare that they have no competing interests.

## Authors' contributions

RD, KG, and WE designed the experiments; RD, KG, JN, SM, and LE performed experiments; KG, GH, KN, and WE provided study material; RD wrote the manuscript, all authors discussed the data and commented on the manuscript draft; all authors read and approved the final manuscript.

## Reviewers' comments

### Reviewers' report 1

Dr. Bhagirath Singh, Department of Microbiology and Immunology, University of Western Ontario, London, Ontario, Canada

In most part this manuscript deals with the immunogenicity of multipotent adult germ-line stem cells (maGSCs) that are derived from spermatogonial stem cells (SSCs) present in adult testis. The authors use these cells as targets for a peptide specific cytotoxic T lymphocytes (CTL) restricted to a major histocompatibility complex (MHC) class I plus OVA (Ovalbumin 257-264) peptide, SIINFEKL.

1) Based upon the data presented the authors conclude that these cells can become targets for CTL even if the expression level of MHC class I molecules is below the detection limit by using flow cytometry. This is not surprising because little as 100 molecules of MHC-peptide complex are sufficient for CTL killing of target cells. Therefore, pluripotent cells might be rejected after transplantation by this mechanism and provides the hope that transplantations of in vitro differentiated cells may be possible and the undifferentiated cells may be killed by their immunogenicity. However, immunologically this approach does not provide any particular benefit as both differentiated cells and undifferentiated cells would be killed in allogeneic conditions. In syngeneic situation it is unclear if there is an advantage to the approach. Authors have to demonstrate this in vivo.

*Authors' response: We indeed agree that it is not surprising that pluripotent stem cells can be killed by CTL. However, contrary data have been reported in the literature *[27]*. Therefore, it was important to demonstrate that pluripotent stem cells are in general not protected against the CTL-mediated cytotoxicity. It remains to be analyzed whether alloreactive CTL that often have lower affinity T cell receptors will similarly kill those targets. Nonetheless, antigens that are specifically expressed in pluripotent teratoma forming stem cells might be relevant targets for CTL and this might be helpful to avoid teratomas after transplantation. However, we completely agree that this is presently a pure speculation that has to be validated or disproved by in vivo experiments in subsequent studies.*

2) I agree with the conclusion that suggests that pluripotent cells are not protected against CTL, even if MHC class I molecules are not detectable by flow cytometry. However, authors should explore this further by RT-PCR analysis for the results to be valid. Similarly the SPI-6 data (Fig. 6) should be confirmed by RT-PCR.

*Authors' response: As suggested, we have analyzed the MHC class I (see new Figure *6*) and SPI-6 expression (see Figure *8*) by quantitative real time PCR.*

3) Finally, it is very important that authors provide a well-balanced analysis to the differences where their results differ from previously published information about the immune-privileged nature of mouse or human ESC as quote in the last paragraph of their Discussion section.

Authors' response: We have altered the discussion in order to clarify these issues.

4) Overall the manuscript is well written and the question posed, methods used are well defined although not new. Additional data is needed to validate the study. The discussion and conclusions need to be refined as outlined above. The title and abstract are well constructed but conclusions need to be modified to identify the shortcomings of the approach and the usefulness of the findings.

Authors' response: We tried also to further clarify the advantages but also the limitations of our present approach in the discussion.

#### Comment to the revised manuscript

The authors have considerably revised and updated the manuscript based on my comments. Additional experiments have been done and added as new figures. Authors have discussed these results in the text and in my view, the revised manuscript provides a more evidence for the observations and conclusions.

Authors' response: We would like to thank Dr. Singh for his thoughtful review of our manuscript and the helpful suggestions for improvement.

### Reviewers' report 2

Dr. Etienne Joly, Equipe de Neuro-Immuno-Génétique Moléculaire, IPBS, UMR CNRS 5089, Toulouse, France

1) Have you considered the possibility that all these pluripotent cells express very low levels of TAP, and have thus very few properly folded MHC molecules on their surface, but probably quite a few empty, peptide-receptive ones. On this subject, I refer you to Bikoff et al. [61] and to my own paper on neuronal cells [40].

*Authors' response: We have analyzed now the expression of Tap1 and Tap2 at the mRNA level (see the new Figure *6*) and found rather low levels in most of the pluripotent stem cell lines. H2K and B2m transcripts were more abundant suggesting that indeed a failure of peptide loading could be responsible for the low MHC class I cell surface expression levels. However, culture at 28°C or in the presence of an H2K^b^-binding peptide did not markedly increase the H2K^b ^cell surface expression (see the new Figure *7*). Thus, it is unlikely that a low TAP expression alone is the reason for the low MHC class I expression on maGSCs and ESCs.*

2) Considering this previous point, and the fact that the effectors in graft rejection would be alloreactive CTL rather than peptide specific ones, I think that you may have found quite a different picture if you had used anti-H2^b ^alloreactive CTLs obtained either after an *in vitro *mixed lymphocyte reaction, or after priming non-H2^b ^mice with H2^b ^splenocytes. Indeed, whereas OT-I cells have very high affinity TCRs that can kill upon recognition of just a handful of peptide-MHC complexes, the TCRs of alloreactive CTLs would be much more likely to be of low affinity, and would thus not have been triggered by the low levels found at the surface of the various stem cells used in your study.

*Authors' response: We agree that using alloreactive CTL might give different results. Therefore, it will be of interest to determine whether the pluripotent cells used in this study can induce an alloreactive immune response and whether they are good targets for alloreactive CTL. However, we think that this question is beyond the scope of the present manuscript. In the present study we wanted to clarify whether the pluripotent cells can in principle be killed by CTL since it has been proposed previously that ESCs are resistant to cytotoxicity mediated by CTL *[27]*. Therefore, we used a well-controlled and very potent system of peptide-specific CTL derived from T cell receptor-transgenic mice instead of less well-defined alloreactive CTL. Using this system we could clearly demonstrate that the targets were killed and that the killing was peptide-dependent. We clarified the advantages but also the possible limitations of our present experimental approach in the discussion.*

3) The last issue is that of the presentation of the FACS data. I find that histograms showing percentages of positive cells or even means of fluorescence are really much less informative than the actual plots. If they all look similar, then you should at least provide one example for each case. And if they are not so similar (for example different shapes), then you should definitely show the plots, and not the percentage of positive cells.

Authors' response: We agree and we have now included representative FACS plots for most of the data in addition to the histograms that summarize several experiments and that are therefore more representative than individual plots.

We would like to thank Dr. Joly for his important and very helpful scientific and editorial suggestions for our manuscript.

### Reviewers' report 3

Dr. Lutz Walter, Department of Primate Genetics, German Primate Center - Leibniz Institute for Primate Research, Göttingen, Germany

This manuscript reports on pluripotent stem cells and their susceptibility to killing by CTL. Pluripotent stem cells include ESCs, iPSCs, and maGSCs. The latter type was examined in detail in this paper. MaGSCs are derived from spermatogonial stem cells after reprogramming via in vitro cultivation. These maGSCs harbour enormous potential in regenerative medicine, as they are able to differentiate into a variety of cell types. Additionally, in an autologous transplantation of differentiated maGSCs no immunologic barrier is expected to occur. Yet, a known problem is formation of teratoma after transplantation of pluripotent cells.

The authors compared teratoma growth in immunodeficient SCID/beige mice after in vitro differentiation in various culture conditions. Differences in teratoma formation were not observed. In contrast, immunocompetent mice were able to reject maGSC and did not develop teratoma, suggesting cytotoxic immune cells mediated killing. These cells usually recognise MHC class I molecules. Therefore, the authors tested expression levels of MHC class I on the surface of different maGSC cell lines, which turned out to be either very low or not detectable, similar to what is known for ESCs. Interestingly, all maGSC lines could be killed by CTL, despite their low or undetectable expression level of MHC class I molecules. As the authors used well-defined CTL from TCR-transgenic OT-I mice, they could demonstrate the peptide-dependency of CTL killing and could exclude NK cell-mediated killing. Furthermore, it was found that maGSCs do not express the serine-protease inhibitor 6, which is known to confer protection against the granule exocytosis effector mechanism of cytotoxicity, providing a potential explanation for the observed CTL killing and the lack of CTL inhibition, which can usually be observed for ESCs.

In summary this represents an interesting and carefully conducted study. I have only minor comments.

**Methods:** The authors should mention the MHC haplotype (H2^b^) of the studied mice and cell lines.

**Results:** On page 14 the expression levels of MHC class I molecules were compared between maGSC Str8 SSC5 cells and RMA. It was concluded that the level for maGSC was low. While I have no doubt that this is true, the question arises whether RMA cells are an adequate control. I guess this cell line was used as a control for antibody binding. Would it be possible to compare the level with spermatogonia or spermatogonial cell lines? At the bottom of page 15, last sentence, the authors should add "and absence of NK-mediated killing."

Authors' response: We would like to thank Dr. Walter for his encouraging review of our work. We have added the information on the MHC haplotype of the studied cell lines in the methods section. The RMA cells were indeed used as positive control for MHC class I expression and antibody binding but also for killing by the CTL. Recently, we have analyzed two newly generated cell lines that represent spermatogonial stem cells (SSCs). Similarly to most maGSCs, they did not express H2K or H2D molecules at the cell surface when analyzed by flow cytometry. However, these cell lines are so far not fully characterized. Therefore, we decided not to present these data in this manuscript. The suggested addition on page 15 has been done.
